# Targeted Therapy of Papillary Thyroid Cancer: A Comprehensive Genomic Analysis

**DOI:** 10.3389/fendo.2021.748941

**Published:** 2021-09-24

**Authors:** Daniel A. Hescheler, Burkhard Riemann, Milan J. M. Hartmann, Maximilian Michel, Michael Faust, Christiane J. Bruns, Hakan Alakus, Costanza Chiapponi

**Affiliations:** ^1^ Department of Nuclear Medicine, University Hospital Münster, Münster, Germany; ^2^ European Institute for Molecular Imaging (EIMI), University of Münster, Münster, Germany; ^3^ Department of General, Visceral, Tumor and Transplant Surgery, University Hospital Cologne, Cologne, Germany; ^4^ Institute of Zoology, University of Cologne Germany, Cologne, Germany; ^5^ Policlinic for Prevention, Diabetes and Endocrinology, University of Cologne, Cologne, Germany

**Keywords:** targeted molecular therapy, new treatment advances, human genome project, thyroid cancer, genomic analysis

## Abstract

**Background:**

A limited number of targeted therapy options exist for papillary thyroid cancer (PTC) to date. Based on genetic alterations reported by the “The Cancer Genome Atlas (TCGA)”, we explored whether PTC shows alterations that may be targetable by drugs approved by the FDA for other solid cancers.

**Methods:**

Databases of the National Cancer Institute and MyCancerGenome were screened to identify FDA-approved drugs for targeted therapy. Target genes were identified using Drugbank. Genetic alterations were classified into conferring drug sensitivity or resistance using MyCancerGenome, CiViC, TARGET, and OncoKB. Genomic data for PTC were extracted from TCGA and mined for alterations predicting drug response.

**Results:**

A total of 129 FDA-approved drugs with 128 targetable genes were identified. One hundred ninety-six (70%) of 282 classic, 21 (25%) of 84 follicular, and all 30 tall-cell variant PTCs harbored druggable alterations: 259 occurred in 29, 39 in 19, and 31 in 2 targetable genes, respectively. The *BRAF* V600 mutation was seen in 68% of classic, 16% of follicular variant, and 93% of tall-cell variant PTCs. The *RET* gene fusion was seen in 8% of classic PTCs, *NTRK*1 and 3 gene fusions in 3%, and other alterations in <2% of classic variant PTCs. Ninety-nine of 128 (77%) FDA-approved targetable genes did not show any genetic alteration in PTC. Beside selective and non-selective *BRAF*-inhibitors, no other FDA-approved drug showed any frequent predicted drug sensitivity (<10%).

**Conclusion:**

Treatment strategies need to focus on resistance mechanisms to *BRAF* inhibition and on genetic alteration–independent alternatives rather than on current targeted drugs.

## Introduction

Papillary thyroid cancer (PTC) is quite rare and has generally a good prognosis: approximately 80% of all cases are cured after radical surgery and radioiodine ablative treatment (RAI). Up to 18% of patients are diagnosed with locoregional recurrence within the first 5 years after initial treatment (1), which is usually cured by a second RAI. Thirty-three to 55% of cases diagnosed with recurrence (2) and approximately 5% of all cases are diagnosed with RAI-refractory (RAI-R) disease (3), which is associated with a significantly poorer outcome (5-year survival <50%).

The European Thyroid Association (ETA) Guidelines state that the choice to continue with RAI therapy in a patient without RAI uptake, with a mixed pattern of uptake or progression despite RAI uptake “can only be addressed in a multidisciplinary team (MDT), weighing the benefits and disadvantages of continuing RAI eventually combined with local treatments” (3). If RAI-R disease is limited to a single lesion or more than one lesion within the same organ, the possibility of performing a local treatment should be considered, according to the ETA (3). Since so far, no systemic therapy option for RAI-R disease is curative, treatment should be started only in the setting of rapid metastatic progress according to recist criteria, taking into account patients’ age and comorbidities.

Chemotherapy plays no significant role in the systemic treatment of advanced differentiated thyroid cancer (DTC). Medical treatment is currently based on tyrosine kinase inhibitors (TKIs) as targeted molecular treatment based on studies showing that cellular dedifferentiation resulting in unresponsiveness to RAI therapy correlate with the degree of MAPK activation (4). The kinase inhibitors Sorafenib and Lenvatinib have been shown to significantly improve progression-free survival (PFS) rates in advanced RAI-R DTCs (4) and have consequently been approved by the Food and Drug Administration (FDA) and the European Medical Agency (EMA) for treatment of advanced RAI-R DTCs (5). An increase in overall patient survival, however, has been described only for Lenvatinib selectively for the subgroup of cases >65 years old (5–7).

There are, however, several drawbacks to PTC treatment with TKIs. The medication needs to be taken for the rest of the patient’s life and has a wide spectrum of adverse side effects with a significant impact on quality of life. Further, some patients develop a resistance to treatment (8). For this reason, it is generally recommended to carefully choose the starting point of treatment, with particular emphasis on the patient’s general condition and quality of life.

Based on *in silico* data mining, we have previously reported that drugs already approved by the FDA for different cancer types show potential to be used in other cancer entities (9). Here, we investigate whether any of the 129 currently FDA-approved drugs act on targets also commonly found in papillary thyroid cancer based on genetic mutations reported by the “The Cancer Genome Atlas (TCGA)”.

## Methods

All data were obtained from open access databases and referenced accordingly. The study was conducted in accordance with the provisions of the Declaration of Helsinki and local laws.

### FDA-Approved Targeted Therapy and Their Biological Targets

We identified all FDA-approved drugs for cancer therapy by searching the databases of the National Cancer Institute (10) and MyCancerGenome (11). Unspecific drugs such as Tretinoin or Cabazitaxel were excluded (**Supplementary Table 1**). One hundred twenty-nine FDA/EMA-approved drugs targeting genetic alterations were included. These drugs were linked to 128 genes by querying the databases of the MD Anderson Cancer Center (12) and Drugbank (13), which include information on the potential sites of binding and action (**Supplementary Table 1**). We further gathered information about specific genetic alterations that confer either drug sensitivity or drug resistance to targeted therapy from the MyCancerGenome (11), CiViC (14), TARGET (15), and OncoKB (16) databases (**Supplementary Table 2**).

### Genetic Alterations in Thyroid Cancer

One of the landmarks of tumor genomics data is “The Cancer Genome Atlas Program”, shortly TCGA. The TCGA data are available through the cBioPortal platform (17, 18), which allows the user to mine the TCGA data on recent insight. The papillary thyroid cancer study (19), listed in cBioPortal namely as “Thyroid Carcinoma (TCGA, Firehose Legacy)”, was chosen as the basis for this study. Mutation data from whole exome sequencing generated by MutSig, putative copy number alterations from GISTIC 2.0, as well as fusion data in papillary thyroid cancer were taken from the supplementary material of the thyroid cancer TCGA study. Only cases with complete datasets of mutation data, putative copy number data, and clinical information about tumor histology were included in this study. The following cancer subtypes were present: 282 patients with classic type PTC, 83 patients with follicular variant PTC, and 30 patients with tall-cell variant PTC (overall n=395). Mutation variants and CNVs directly or indirectly affecting genes that have been targeted by FDA-approved drugs were identified.

### Drug Response Prediction

The genetic datasets of these 395 patients were mined for (a) Gain of function, (b) CNV-Amplification, (c) gene fusion, or (d) specific genetic alterations that could potentially confer drug response to a given targeted therapy.

### Gain of Function

Gene alterations resulting in gain of function were annotated according to OncoKB (16) and Civic (14). Activating gene alterations were annotated with OncoKB’s (like) gain of functions, Civic clinical significance score of “(Likely) pathogenetic” or positive, as well as whether the alteration was in a hotspot as defined by Chang et al. (20).

### CNV Amplification

The data from cBioPortal (17, 18) is annotated with a copy number analysis algorithm [GISTIC 2.0 (21)], which indicates the copy number level per gene: “− 2” deep loose, “− 1” shallow loose, “0” diploid, “1” low-level gain, and “2” high-level amplification. The threshold of high-level amplification “2” was chosen, to signify an occurrence of a copy number increase in each tissue sample.

### Specific Gene Alterations

Each drug shows a literature-based effectiveness upon a specific alteration found in TCGA tissue as well as an indirect gene alteration affecting resistance or sensitivity to a drug. For this we utilized MyCancerGenome (11), CiViC (14), TARGET (15) and OncoKB (16).

### Mutation Hotspot Analysis

Mutation variants known to be responsive to FDA-approved drugs were searched according to the database DOCM (22).

### Pathway Analysis

A pathway analysis was performed using PathwayMapper through cBioPortal.

### *BRAF* in PanCancer Cohorts

The 395 cases of PTC as well as all TCGA-PanCancer cohorts (33 cancer cohorts, total 11,084 cases) were screened for *BRAF* alterations in cBioportal.

## Results

### Genetic Alterations in Targeted Genes

One hundred twenty-eight targetable genes of 129 FDA-approved targeted therapies were identified. There were 259 alterations in 29 drug-targetable genes in classic PTC, 39 in 19 druggable genes in follicular variant of PTC, and 31 in 2 druggable genes of tall-cell variant PTC. One hundred ninety-six (70%) of 282 classic variant, 21 (25%) of 84 follicular variant, and all 30 (100%) tall-cell variant PTC tumors harbored druggable mutations.

**Figure 1:**
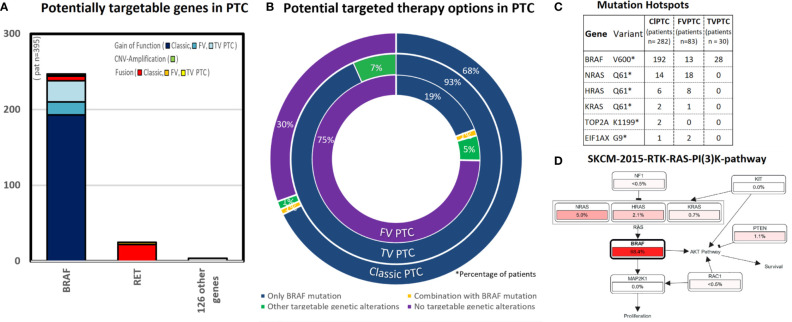
This figure shows the results of potential targetable genetic alterations in papillary thyroid cancer [total 395 cases PTC, respectively subtypes of papillary thyroid cancer as follows: classic type PTC (282 cases), follicular variant PTC (83 cases), and tall-cell variant PTC (30 cases)]. **(A)** This bar chart lists the number of cases (y-axis) with either gain of function mutation (blue colors), CNV amplification (green), and others (red colors). Almost all targetable genetic alterations occur in the gene BRAF. **(B)** The triple doughnut chart shows the potential targeted therapy options for classic type PTC (outer circle), tall-cell variant PTC (middle circle), and follicular thyroid cancer (inner circle). BRAF inhibitors are potential treatable in 68% cases of classic type PTC. In comparison, 75% cases of the follicular variant PTC had no targetable alterations. **(C)** The point mutation BRAF V600 occurs in 68% cases in classic type PTC, 16% cases in follicular variant PTC, 93% cases in tall-cell variant PTC. Interestingly, other mutation hotspots in classic type PTC occurred, as well in the RAS-pathway as follows: NRAS (5%, 14/282 cases) and HRAS (2%, 6/282 cases). **(D)** Pathway analysis [created by Pathway Mapper (61) through cBioportal (18)]: In the pathway analysis for classic-type papillary thyroid cancer, we identified a high match in the SKCM-2015-RTK-RAS-PI(3)K-pathway, especially in the RAS-signaling pathway (BRAF,HRAS,NRAS).

Of all targetable alterations, only the *BRAF* V600 variant shows a high frequency in the classic type of PTC (68%, 192/282 cases), 93% (28/30) of cases in tall-cell variant PTC, and 16% (13/83) of cases with follicular variant PTC (see **Figure 1A**). The specific *BRAF* mutation is nearly exclusively *BRAF* V600E (192/193 “*BRAF*-mutated cases” in classic types of PTC). All cases of tall-cell variant PTC showed at least one *BRAF* alteration [93% (28/30) cases with a *BRAF*-mutation and 7% (2/30) cases with a *BRAF* gene fusion]. This is followed by *RET* gene fusion, which occurs in 8% of cases with classic type PTC, and *NTRK*1 and 3 gene fusions, which occur in 3% of cases with classic type PTC. All other genetic alterations are rare (<2%, see **Figure 1B**). We found that 99 potentially targetable genes did not show alterations such as CNV amplification, gene fusion, or gain of function mutations in the PTC cases we analyzed.

### Mutation Hotspot Analysis

The highest frequency mutation hotspot in classic type PTC occurs in the point mutation *BRAF* V600E (68% cases). The second and third highest frequency of mutation hotspots occurred also in the RAS-Pathway: *NRAS* Q61 (5%, 14/282 cases) and *HRAS* Q61 (2%, 6/282 cases) (see **Figure 1C**). Moreover *NRAS Q61K* is a predictive biomarker in three National Comprehensive Cancer Network (NCCN) guidelines in at least one clinical setting for binimetinib, cetuximab, and panitumumab (24).

### Pathway Analysis

We identified a match in the SKCM-2015-RTK-RAS-PI (3)K-pathway for the RAS-Signaling pathway (*BRAF*, *HRAS*, *NRAS*) as shown in the **Figure 1D**. Pathways other than RAS- and RET are rare in PTC (<1%).

### Potential Drug Option

**Figure 2:**
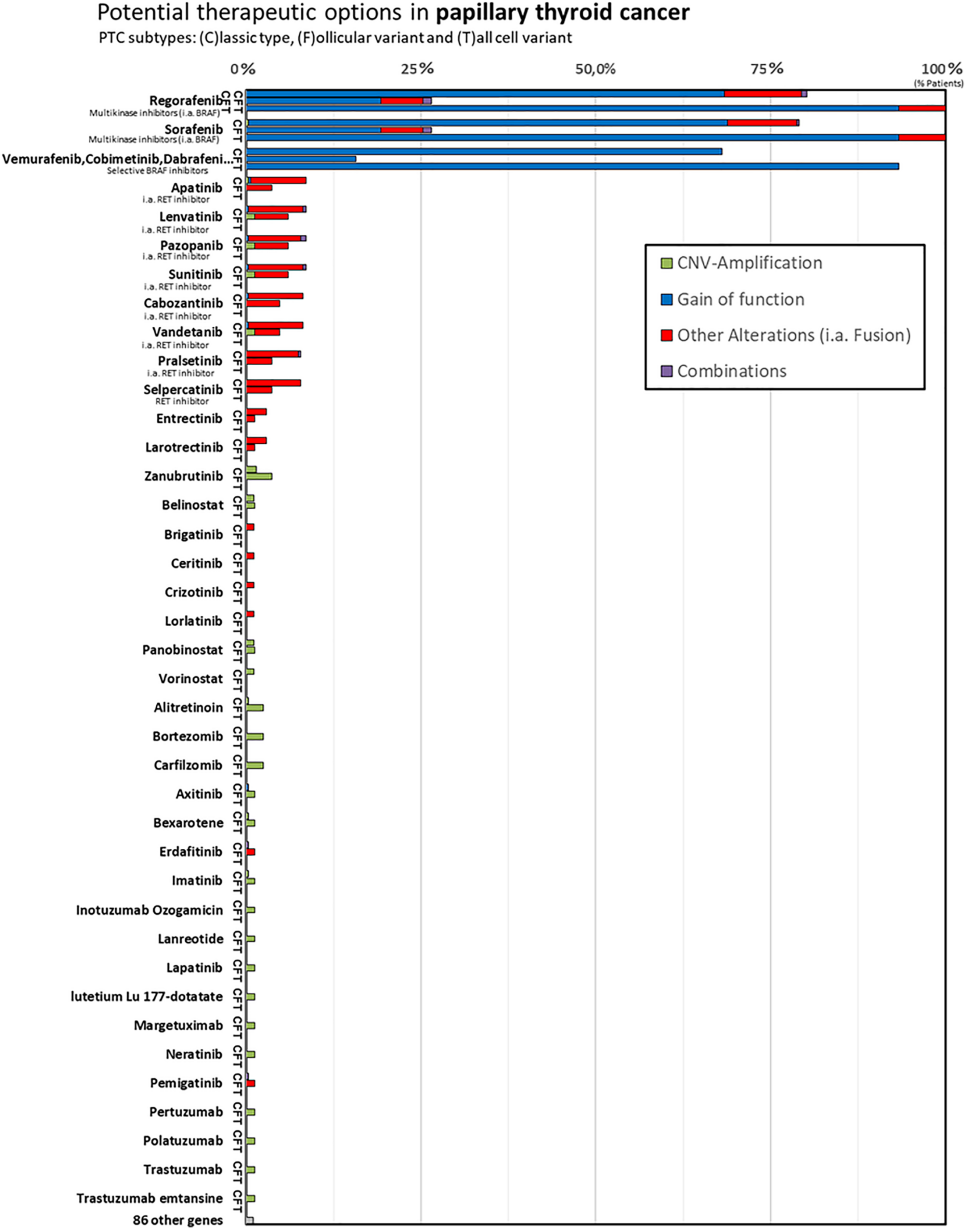
In the column chart y-axis lists the FDA-approved cancer drugs and the x-axis shows the number of cases with (c)lassic type PTC (shortly C), follicular variant PTC (shortly F), tall-cell variant PTC (shortly T). In our *in silico* analysis we identified BRAF inhibitors (selective BRAF inhibitors or multikinase, i.a., BRAF inhibitors) to be genetically predicted for drug response in papillary thyroid cancer. In comparison, the “non-BRAF inhibitors” show no or low genetically predicted drug response in differentiated thyroid cancer, although Lenvatinib, for example, represents first-line treatment for radioiodine refractory DTC currently.

In our *in silico* analysis, *BRAF* inhibitors (selective *BRAF* inhibitors or multikinase *BRAF* inhibitors) were identified as potential drugs that would lead to a drug response in classic type PTC and tall-cell variant PTC based on the alterations we found. The multikinase *BRAF* inhibitors Regorafenib and Sorafenib showed a response prediction in all tall-cell variant, in >75% classic variant, and in >25% follicular variant PTC. The selective *BRAF* inhibitors Vemurafenib, Cobimetinib, and Dabrafenib displayed a response prediction in 93% of tall-cell variant, in 68% of classic variant, and in 15% of follicular variant PTC. The 122 remaining “non-*BRAF* inhibitors” show no or low (under 10% cases) genetically predicted drug response in PTC (see **Figure 2**).

### Focus on *BRAF*

**Figure 3:**
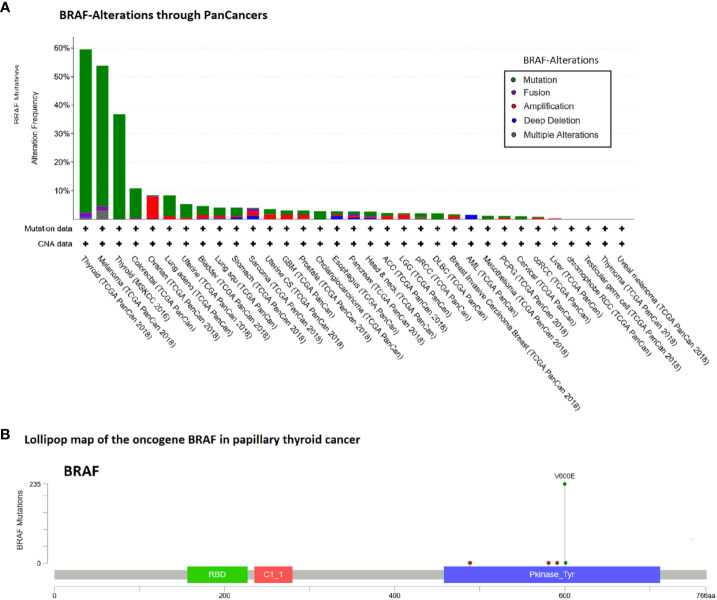
Alteration frequency analysis of BRAF gene in human cancers and lollipop map of BRAF mutation distribution using cBioPortal (18). **(A)** In the TCGA-panCancer cohorts besides the thyroid cancers, the high frequency of BRAF alterations occurs in the Melanoma TCGA-cancer cohort (53.8% of 444 cases). **(B)** Lollipop map of the oncogene BRAF shown are the case number of all variant types of papillary thyroid cancer (60%, 236/395 cases).

The data above suggest that the point mutation *BRAF* V600E possibly plays a pathognomonic role in papillary thyroid cancer. Consequently, we analyzed *BRAF* alterations in the TCGA-panCancer cohorts. A high frequency of *BRAF* alterations besides the thyroid cancers occurs in the “Melanoma TCGA-cancer” cohort (54% of 444 cases) (see **Figures 3A, B**).

## Discussion

Current targeted therapy options for radioiodine-refractory PTC are based on data from placebo-controlled randomized trials in the absence of sufficient clinical trial data comparing single agents or combinations (25). The current options encompass either mutation-specific or antiangiogenic multitargeted kinase inhibitors (aaMKIs). They frequently produce objective response, but no general prolongation of overall survival, and they have significant toxicities (4–7).

In this study we attempted to find new targetable options based on genes commonly altered in PTC. We analyzed the TCGA thyroid cancer study for commonly affected genes and found a high mutation rate in only one single point mutation: the *BRAF* V600 mutation. This mutation is seen in 68% of classic papillary thyroid cancer and in 93% of tall-cell variant patients. Given the predominance of *BRAF* mutations in RAI-R DTC (26, 27), it is possible that classic papillary thyroid cancer and tall-cell variant more often develop to refractory disease. The second highest frequency of mutation hotspots occurred in the RAS-Pathway. *NRAS*, *HRAS*, and *KRAS* mutations, which are seen in 30–50% of follicular thyroid cancer (FTC) (28), were detected in 8% of classic and in 33% of follicular variant PTCs. *BRAF* and RAS mutation are mutually exclusive in DTC (19). An association between the type of mutation (*BRAF* or RAS) and the metastasis pathway typical for papillary and follicular thyroid cancer (lymphogenic or hematogenic) has also been described (29). Although the RAS-Pathway displayed the second highest frequency mutation hotspots, there are no FDA-approved drugs targeting the specific mutations found in *NRAS*, *HRAS*, and *KRAS* for PTC. At this time, it is unclear if drugs targeting *NRAS*, *HRAS*, or *KRAS* (BI1701963 targeting *KRAS* and Tipifarnib targeting *HRAS*) will have any relevance for PTC in the future. Ho et al. (30) reported stable disease in 9 of 11 *HRAS*-mutated thyroid cancer patients treated with Tipifarnib; however, they focus on head and neck squamous cell carcinoma in their report and the specific histology of the thyroid cancer patients is not included. Given the predominance of RAS mutations in RAI avid as opposed to RAI-refractory DTC reported in the literature (31), this pathway might be less relevant for the treatment of RAI-R disease. Other potential targetable genetic alterations were found in less than 10% cases: the *RET* gene fusion was found in 8%, *NTRK*1 and 3 gene fusion in 3%, and other mutations in less than 2% of classic papillary thyroid cancers.

Accordingly, our *in silico* analysis predicted a high drug response for both the multikinase *BRAF* inhibitors (Regorafenib and Sorafenib) and the selective *BRAF* inhibitors (Dabrafenib, Vemurafenib, Cobimetinib), particularly in classic and tall-cell PTC (**Figure 2**). These results may not be surprising. Sorafenib has been shown to significantly improve median progression free survival (PFS) compared to placebo in patients with RAI-refractory DTC (10.8 *vs.* 5.8 months, p < 0.0001) in the phase 3 DECISION trial (32), and it is one of the two currently recommended first-line treatment agents. Regorafenib is very similar to Sorafenib in its molecular structure, except for the addition of a fluorine to the center phenyl ring. Regorafenib shows increased anti-angiogenic activity over Sorafenib (33). Studies on medullary thyroid cancer are currently recruiting (NCT02657551). However, there is currently no data or studies on Regorafenib in radioiodine-refractory papillary thyroid cancer (33).

Interestingly, our study only predicted a low drug response for the other first-line agent Lenvatinib. Our *in silico* analysis predicted a drug response of Lenvatinib similar to that predicted for Apatinib, Pazopanib, Sunitinib, Cabozatinib, Vandetanib, Pralsetinib, and Selpercatinib. However, Lenvatinib has not only been shown to significantly improve progression-free survival (PFS) rates in advanced RAI-R DTCs similar to Sorafenib (4), but it also represents the only current targeted therapy, for which an increase in overall survival has been shown in the >65 year old subgroup (5–7). In the SELECT study, the benefit of Lenvatinib was independent of the *BRAF* and *RAS* mutational status (34). A possible reason is the wide range of tumor cell targets affected (*VEGF*, *VEGFR*1–3, *FGFR*1–4, *PDGFR*α, *KIT*, and *RET*) (35). Studies on Apatinib (NCT03048877), Pazopanib (NCT01813136), Sunitinib (NCT00519896), and Cabozantinib (NCT03690388) on radioiodine-refractory thyroid cancer are ongoing (**Table 1**).

**Table 1:** Overview of BRAF/MEK Inhibitors in clinical trials in differentiated thyroid cancer [modified from Pottier et al. (36)].

Drug/ClinicalTrials.gov	Enrolled Cases	Primary Outcome	Study Design	Results	Reported Adverse Events
Sorafenib (32)	DTC: 209 (locally advanced or metastatic RAI refractory disease)	PFS	Phase III, two arms, randomized, double blinded, placebo controlled (decision trial)	PFS: 10.8 months *vs.* 5.8 for placebo (regardless of mutation status) PR: 12%	Hand–foot skin reaction, diarrhea, alopecia, skin rash or desquamation
Sorafenib (37)	DTC: 16, MTC: 15, ATC: 3 (metastatic progressive unsuitable for surgery, RAI, or radiotherapy)	ORR	Retrospective, Spanisho_-label-sorafenib-use program	DTC PR: 19%	Hand–foot skin reaction, diarrhea, alopecia, skin rash or desquamation
				MTC PR: 47%	
				ATC PR: 33%	
Dabrafenib (38)	DTC: 13 (BRAFV600E mutant disease)	ORR	Subset of phase I study	PR: 29%	Skin papilloma hyperkeratosis, alopecia, fatigue, fever, diarrhea
Trametinib OR Dabrafenib NCT03244956	DTC RAI refractory with RAS (trametinib) or BRAFV600E (dabrafenib) mutation	ORR	Phase II, two arms, open label, recruiting	N/A Estimated End Date 12/2022	
Trametinib/combination Dabrafenib and Trametinib or Vemurafenib and Cobimetinib (39)	DTC: 6 (3 BRAFV600E positive treated with combination, 3 NRAS-positive treated with trametinib)	Restoration of RAI uptake	Retrospective, cohort study	RAI uptake restoration: BRAFV600E (3/3), NRAS (1/3) with median follow-up 16.6 months	Cobimetinib: diarrhea, pyrexia, photosensitivity reaction, abnormal LFT, hyponatremia
Trametinib with RAI NCT02152995	DTC: RAS mutant or RAS/RAF wild-type RAI-refractory recurrent and/or metastatic disease	PFS	Phase II, single arm, open label, recruiting	N/A Estimated End Date 12/2020 (clinicaltrials.gov)	Acneiform rash
Vemurafenib (40)	DTC: 51 (unresectable and metastatic RAI refractory BRAFV600E mutant disease)	ORR	Phase II, parallel assignment, open label	PR 38.5% (VEGFR multikinase inhibitor naïve cohort) PR 27% (prior treatment with VEGFR multikinase inhibitors)	Rash, fatigue, arthralgia

The table lists the clinical trials on BRAF/MEK Inhibitors in differentiated thyroid cancer in terms of study design, primary outcomes, and reported adverse events. The phase III study by Brose et al. (32) showed promising results for Sorafenib (PFS 10.8 months for Sorafenib-arm against 5.8 for placebo), as well as the phase I studies [Falchook et al. (38) and Brose et al. (32)] showed 29% partial remission for Dabrafenib and 38.5% PR for Vemurafenib. In the supplementary file, “Clinical trials in DTC” lists 43 clinical trials of BRAF and other inhibitors in differentiated thyroid cancer.

The V600 mutant specific inhibitors Vemurafenib (PLX4032) and Dabrafenib (GSK2118436) for which the *in silico* analysis predicts a high drug response and which have produced excellent response rates in melanoma studies and in hairy cell leukemia (41) are typically reserved for patients who show a contraindication for first-line therapy with multikinase inhibitors and are administered in off-label use. Although objective responses and a prolongation of progression-free survival in small phase 2 trials were seen, these results have not yet been demonstrated in larger randomized trials (40, 42–51) (**Table 1**). Consequently, the inhibitors haven’t been approved by the FDA for thyroid cancer because toxicity seems to exceed the oncologic benefit. However, like Cobimetinib (GDC0973) (39), Vemurafenib and Dabrafenib have been reported to allow re-expression of genes responsible for iodine metabolism, restoring radioiodine uptake and permitting subsequent radioiodine treatment. In a trial with 10 *BRAF* V600 mutated PTC cases, 60% revealed new radioiodine uptake after 4 weeks of Dabrafenib therapy. Three months after radioiodine therapy in two of the six cases, a partial response could be observed (52). A similar partial response was also described for combined treatment with Cobimetinib and Vemurafenib in *BRAF* V600 mutated PTC cases by Iravani et al. (39). More data on the use of these inhibitors in PTC are critically needed, and “redifferentiation” is so far no clinical standard.

Given the fact that clinical trials with *BRAF* inhibitors produced various outcomes in different carcinomas, we performed a pan-cancer analysis for *BRAF* mutations (**Figures 3A, B**). A detailed knowledge and understanding of these results may also offer insights in the biology of PTC. The contrasting results of V600 mutant specific inhibitors in thyroid cancer compared to melanoma and hairy cell leukemia (53) could be due to different response and resistance mechanisms to *BRAF* inhibition dependent on cell types. A similar contrast was observed in clinical trials for colorectal and non-small-cell lung cancer compared to malignant melanoma (54). The mechanism responsible for a poor response could, for example, include the inhibition of the BCL2 pathway, a concurrent *PI3KCA* mutation and secondary alterations in the MAPK pathway (55). In colon carcinoma, for example, *BRAF* V600E inhibition has been shown to cause a rapid feedback activation of *EGFR*, which supports continued proliferation in the presence of *BRAF* V600E inhibition (56). Melanoma cells instead express low levels of *EGFR* and are therefore not subject to this feedback activation. Similarly co-treatment of thyroid cancer cell lines with an *EGFR* inhibitor increased antitumor efficacy and suppressed resistance to the *BRAF* V600E inhibition (57, 58).

Because the currently recommended first-line agents Lenvatinib and Sorafenib lack specificity for *BRAF* and the toxicity profile still represents a clinical hurdle, other treatment options would be useful in adjuvant setting after surgical management of radioiodine-refractory relapse. Since radioiodine-refractory papillary thyroid cancer is rare (3% of patients) and targeted therapy is currently initiated only after disease progression in a palliative setting, the present study was performed to select the most promising candidates among FDA drugs approved for solid cancer. The major limitation in this study is the fact that this is an *in silico* analysis. Clinical data would be critical to verify the results presented. Secondly, all data presented are based on the TCGA dataset and, thus, on primary papillary instead of radioiodine-refractory thyroid cancer. However, Sabra et al. (31) described *BRAF* mutations as predominant in RAI-R DTC and *RAS* mutations in radioiodine-avid DTC (19). For this reason, the results presented can possibly be extended to radioiodine-refractory thyroid cancer. It also needs to be mentioned that beside the histological subtype, other parameters such as pTNM stage, the patient’s age or co-morbidities must be considered for treatment stratification. In addition, spatial and temporal tumor heterogeneity, which are particularly relevant for the treatment of advanced thyroid cancer (59–61), are not sufficiently represented by the TCGA data. In the era of personalized medicine, the answer will likely require an ultra-deep sequence of the patient’s primary tumor in an attempt to identify relevant mutated genes. Appropriate drug combinations will help target the spatial and prevent the temporal heterogeneity of tumors, with the eradication of these clones (62). In addition, it is unclear how the data presented relates to pediatric patients, as the TCGA dataset does not cover patients <18 years. It is likely that the majority of pediatric PTC patients with high-risk tumors have a high genetic component and therefore druggable targets (63, 64).

Finally, several studies have demonstrated that *TERT* promoter mutations, particularly in association with *BRAF* V600E, correlate with negative clinical characteristics such as RAI- refractory disease (27, 65). *TERT* promoter mutations were reported in 36 (9.4%) of 384 informative tumors in the TCGA data (19). Further, for distant metastases, an enrichment in *TERT* mutations has been described (66). There are currently studies on telomerase inhibitors (INO5401, Telomelysin, and Imeltestat) on myeloid malignancy. These drugs might also play a role in the future for the treatment of RAI-R disease.

In summary our *in silico* analysis for drug prediction response based on alterations described in the TCGA data suggested *BRAF* inhibitors as the most promising target for most classic and tall-cell variants of thyroid cancer. The multikinase *BRAF* inhibitor Sorafenib is currently the first-line treatment for radioiodine-refractory thyroid cancer; however, for Regorafenib, clinical data for differentiated thyroid cancer are still lacking; and for the selective *BRAF* inhibitors (Dabrafenib, Vemurafenib, Cobimetinib), insufficient evidence has been collected for FDA approval in PTC treatment. Similar to colorectal and small lung cancer, resistance mechanisms might be responsible for the decreased efficacy of these medications. This needs to be considered in future studies. For Lenvatinib, which plays a major role in first-line targeted therapy of radioiodine-refractory thyroid cancer, our *in silico* analysis predicts a low response. Since targeted therapy does not lead to a significant improvement of overall survival in younger patients and is associated with high toxicity, alternative treatment options are urgently needed. These data suggest that resistance mechanisms to *BRAF* inhibition should be further investigated in order to ameliorate drug responses in the future.

## Data Availability Statement

The original contributions presented in the study are included in the article/**Supplementary Material**. Further inquiries can be directed to the corresponding author.

## Author Contributions

DH and HA designed the computational model and framework. DH, HA, and CC carried out the implementation. DH, BR, MH, MF, CB, HA, and CC contributed to the interpretation of the results. DH, BR, MH, MM, MF, CB, HA, and CC contributed critical feedback and helped shape the research, analysis, and manuscript. CC and DH wrote the first draft of the manuscript, and all authors critically revised the manuscript. All authors contributed to the article and approved the submitted version. All authors decided to submit this study and agreed to be accountable for all aspects of the work as recommended by the “International Committee of Medical Journal Editors” (ICMJE) authorship criteria.

## Conflict of Interest

The authors declare that the research was conducted in the absence of any commercial or financial relationships that could be construed as a potential conflict of interest.

## Publisher’s Note

All claims expressed in this article are solely those of the authors and do not necessarily represent those of their affiliated organizations, or those of the publisher, the editors and the reviewers. Any product that may be evaluated in this article, or claim that may be made by its manufacturer, is not guaranteed or endorsed by the publisher.
