# Supplementary material for: Targeted Therapy of Papillary Thyroid Cancer: A Comprehensive Genomic Analysis
Source: Front Endocrinol (Lausanne). 2021 Sep 24;12:748941. doi: 10.3389/fendo.2021.748941 (PMC8498581; doi:10.3389/fendo.2021.748941)
Supplement: Supplementary file 2 [file Table_2.docx]

**Table: Clinical trials in differentiated thyroid cancer (DTC) adopted and modified from Al-Jundi et al.** (1).

| **Drug/**  **ClinicalTrials.gov**  **ID/Reference** | **Mechanism of Action** | **Enrolled Patients *** | **Primary Outcome** | **Study Design** | **Results** | **Reported Adverse Events** |
| --- | --- | --- | --- | --- | --- | --- |
| **Tyrosine Kinase Inhibitors** | | | | | | |
| Anlotinib  NCT04309136 | VEGFR, PDGFR, FGFR1 | DTC/MTC (locally advanced  thyroid cancer with/without  distant metastasis) | ORR | Phase II, single arm, open label,  recruiting | N/A  Estimated End Date 6/2022  (clinicaltrials.gov) | Hand–foot syndrome, lipid  profile abnormalities, fatigue,  diarrhea, proteinuria |
| Anlotinib  NCT02586337 |  | DTC (RAI refractory disease) | PFS | Phase II/III, two arms,  randomized, double blinded,  placebo controlled, active,  not recruiting  (ALTER01032) | N/A  Estimated End Date 12/2019  (clinicaltrials.gov) |  |
| Apatinib  NCT03048877 | VEGFR | DTC (locally advanced or  metastatic disease) | PFS | Phase III, two arms,  Randomized, double blinded,  placebo controlled, active,  not recruiting | N/A  Estimated End Date 6/2021  (clinicaltrials.gov) | Hypertension, hand–foot  syndrome, proteinuria, fatigue |
| Apatinib  NCT03167385 |  | DTC (locally advanced or  metastatic disease) | Disease control rate | Phase II, single arm,  open label, recruiting | N/A  Estimated End Date 12/2020  (clinicaltrials.gov) |  |
| Axitinib  Cohen et al.[2] | VEGFR, PDGFR, KIT | DTC: 45 (resistant to or not  appropriate for RAI)  MTC: 11  ATC 11 | ORR | Phase II, single arm, open label | ORR of 30%  SD for _ 16 weeks: 38%  PFS: 18.1 months | Fatigue, diarrhea, nausea,  anorexia, hypertension,  stomatitis |
| Axitinib  Capdevila et al. [3] |  | DTC: 34 (RAI refractory)  MTC: 13 | ORR | Retrospective study,  compassionate use program  (CUP) in Spain | ORR for PTC: 29.4%  PFS for PTC: 7.4 months  ORR for MTC: 23.1%  PFS for MTC: 9.4 months |  |
| Cabozantinib NCT03690388 | VEGFR, RET, MET, FLT3,  AXL | DTC (RAI disease that  progressed after use of  VEGFR–TKI therapy) | PFS  ORR | Phase III, two arms,  randomized, double blinded,  placebo controlled, recruiting | N/A  Estimated End Date 12/2022  (clinicaltrials.gov) | Diarrhea, hand–foot syndrome,  weight loss, decreased appetite,  nausea, fatigue |
| Cabozantinib NCT02041260 |  | DTC (RAI refractory disease  with radiographic progression  in the past 14 months | Number of AEs | Phase II, single arm, open label,  active, not recruiting | N/A  Estimated End Date 3/2021  (clinicaltrials.gov) |  |
| Donafenib  NCT03602495 | VEGFR | DTC (RAI refractory/resistant  disease) | PFS | Phase III, two arms,  randomized, double blinded,  placebo controlled, recruiting | N/A  Estimated End Date 3/2021  (clinicaltrials.gov) | Hand–foot syndrome, diarrhea,  rash, hair loss, hypertension,  tachycardia |
| Dovitinib  Lim et al. [4] |  | DTC: 28 (RAI refractory or  not appropriate)  MTC: 12 | ORR | Phase II, single arm, open label | ORR: 20.5%  Median PFS: 5.4 months | Diarrhea, anorexia, nausea,  vomiting, fatigue |
| Lenvatinib  Schlumberger et al. [5] | VEGFR, PDGFR, EGFR,  RET, KIT | DTC: 261 (RAI refractory  progressive disease) | PFS | Phase III, two arms,  randomized, double blinded,  placebo controlled | Median PFS: 18.3 vs. 3.6 for placebo  ORR: 64.8% (CR 4/261 + PR 165/261) | Hypertension, diarrhea,  fatigue, anorexia, weight loss,  nausea |
| Lenvatinib  NCT03573960 |  | DTC (locally recurrent or  metastatic progressive RAI  refractory disease) | -Grade 3 and higher  TEAEs  -Number of dose  reductions  -Time to 1st dose  reduction | Phase IV, single arm, open  label, recruiting | N/A  Estimated End Date  12/2020  (clinicaltrials.gov) |  |
| Lenvatinib  NCT03506048 |  | DTC (progressive despite RAI  in the past 12 months | Time to progression | Phase II, single arm, open label,  recruiting | N/A  Estimated End Date  1/2021  (clinicaltrials.gov) |  |
| Lenvatinib  NCT02702388 |  | DTC (RAI refractory disease) | -PFS  -TEAEs | Phase II, two arms,  randomized, double blinded,  evaluating the starting dose  18 mg vs. 24 mg, active,  not recruiting | N/A  Estimated End Date 9/2020  (clinicaltrials.gov) |  |
| Lenvatinib  NCT02966093 |  | DTC (RAI refractory disease in  China) | PFS | Phase III, two arms,  randomized, double blinded,  placebo controlled, active,  not recruiting | N/A  Estimated End Date 4/2021  (clinicaltrials.gov) |  |
| Nintedanib  NCT01788982 | VEGFR, PDGFR, FGFR,  RET, FLT, | DTC  MTC  (as second-line therapy if  progressive disease after  first-line therapy) | PFS | Phase II, two arms,  randomized, double blinded,  active not recruiting | N/A  Estimated End Date  9/2019  (clinicaltrials.gov) | Diarrhea, nausea, vomiting,  abdominal pain |
| Pazopanib  Bible et al. [6] | VEGFR, FGFR, PDGFR,  RET, KIT | DTC: 37  (progressive RAI  refractory disease) | Tumor response  rate | Phase II, two arms, open label | PR: 49% | Fatigue, skin and hair  hypopigmentation, diarrhea,  nausea |
| Sorafenib  Brose et al. [7] | VEGFR, PDGFR, RET, KIT,  FLT | DTC: 209 (locally advanced or  metastatic RAI  refractory disease) | PFS | Phase III, two arms,  randomized, double blinded,  placebo controlled  (decision trial) | PFS: 10.8 months vs. 5.8 for placebo  (regardless of mutation status)  PR: 12% | Hand–foot skin reaction,  diarrhea, alopecia, skin rash  or desquamation |
| Sorafenib  Capdevila et al. [8] |  | DTC: 16  MTC: 15  ATC: 3  (metastatic progressive  unsuitable for surgery, RAI,  or radiotherapy) | ORR | Retrospective, Spanish  o_-label-sorafenib-use  program | DTC PR: 19%  MTC PR: 47%  ATC PR: 33% |  |
| Sunitinib  Bikas et al. [9] | VEGFR, PDGFR, RET, KIT,  FLT | DTC: 23 (metastatic, residual,  recurrent,  or progressive disease) | ORR | Phase II, single arm, open label,  as adjunctive treatment | PR: 26%  SD: 57% | Cytopenia, diarrhea, fatigue,  hand–foot skin reaction,  nausea, musculoskeletal  pain, hypertension |
| Sunitinib  Carr et al. [10] |  | DTC: 28 (RAI refractory  disease,  FDG–PET avid disease)  MTC: 7 (FDG–PET  avid disease) | ORR | Phase II, Single Arm, Open  Label | DTC ORR: 28%  MTC ORR: 50% |  |
| Sunitinib  Ravaud et al. [11] |  | DTC: 41 (RAI resistant)  MTC: 26  ATC: 4  (sunitinib as a first-line  anti-angiogenic therapy) | ORR | Phase II, single arm, open label | DTC PR: 22%  MTC PR: 38.5%  ATC: no response |  |
| Vandetanib  Leboulleux et al. [12] | VEGFR, EGFR, RET, KIT | DTC: 72 (locally advanced or  metastatic disease) | PFS | Phase II, randomized,  double blind,  placebo controlled | PFS 11.1 vs. 5.9 months for placebo | Diarrhea, rash, nausea, hypertension, headache |
| Vandetanib  NCT01876784 |  | DTC: 119 (locally advanced or  metastatic RAI refractory or  unsuitable disease) | PFS | Phase III, two arms,  randomized, double blind,  placebo controlled | PFS (no statistically significant  di_erence—10.0 months vs.  5.7 months with p value 0.080) |  |
| **BRAF Inhibitors** | | | | | | |
| Dabrafenib  Falchook et al. [13] | BRAFV600E | DTC: 13 (BRAFV600E  mutant disease) | ORR | Subset of phase I study | PR: 29% | Skin papilloma hyperkeratosis,  alopecia, fatigue, fever,  diarrhea |
| Vemurafenib  Brose et al. [14] | BRAFV600E | DTC: 51  (unresectable and metastatic  RAI refractory BRAFV600E  mutant disease) | ORR | Phase II, parallel assignment,  open label | PR 38.5% (VEGFR multikinase  inhibitor naïve cohort)  PR 27% (prior treatment with  VEGFR multikinase inhibitors) | Rash, fatigue, arthralgia |
| **Radioactive Iodine Restoration Treatments** | | | | | | |
| Selumetinib  Ho et al. [15] | MEK1, MEK2 | DTC: 20  (RAI refractory  disease—9 BRAF mutation,  5 NRAS mutation) | The percentage of  patients with  selumetinib-induced  increases in iodine  uptake in the  index tumor | Open label, single arm,  treatment with selumetinib,  then evaluate by RAI  uptake study | Increased I-124 uptake in 12/20  (4/9 with BRAF mutations)  (5 of 5 with NRAS mutations)  (8/12 reached dosimetry threshold  for RAI treatment) | Fatigue, maculopapular rash,  elevated liver enzymes,  acneiform rash |
| Selumetinib  ISRCTN17468602 (UK) |  | Locally advanced and  metastatic RAI refractory DTC | PFS | Phase II, single arm,  treatment with selumetinib,  then evaluate by RAI  uptake study  (SEL-I-METRY Trial) | N/A |  |
| Trametinib with RAI  NCT02152995 | MEK1, MEK2 | DTC: RAS mutant or RAS/RAF  wild-type RAI-refractory  recurrent and/or  metastatic disease | PFS | Phase II, single arm, open label,  recruiting | N/A  Estimated End Date  12/2020  (clinicaltrials.gov) | Acneiform rash |
| Trametinib OR Dabrafenib  NCT03244956 |  | DTC RAI refractory with RAS  (trametinib) or BRAFV600E  (dabrafenib) mutation | ORR | Phase II, two arms, open label,  recruiting | N/A  Estimated End Date  12/2022 | See Trametinib  See Dabrafenib |
| Trametinib/  combination Dabrafenib and  Trametinib or Vemurafenib and  Cobimetinib  Irvani et al. [16] | Trametinib (see above),  Dabrafenib (see above),  Vemurafenib (see above),  Cobimetinib: MEK1, MEK2 | DTC: 6 (3 BRAFV600E positive  treated with combination,  3 NRAS-positive treated  with trametinib) | Restoration of RAI  uptake | Retrospective, cohort study | RAI uptake restoration:  BRAFV600E (3/3), NRAS (1/3) with  median follow-up 16.6 months | See Trametinib  See Dabrafenib  See Vemurafenib  Cobimetinib: diarrhea, pyrexia,  photosensitivity reaction,  abnormal LFT, hyponatremia |
| **Peptide Receptor Radionuclide Therapy** | | | | | | |
| PRRT  90Y-DOTATATE or  177Lu-DOTATATE  Budiawan et al. [17] |  | DTC: 7  MTC: 8  Mixed DTC and MTC: 1  (non-RAI avid and RAI  refractory thyroid cancer) | Treatment response  Treatment  related toxicity | Phase II trial, single arm,  open label | PR 18.2%  SD 36.4%  Median PFS 25 months  Mean survival 4.2 years | Mild hematological toxicity,  abnormal liver enzymes,  mild nephrotoxicity |
| PRRT  (90)Y-DOTATOC  Versari et al. [18] |  | DTC: 41 (RAI negative enrolled  in the study 11/41 patients  were treated with PRRT) | Treatment response | Phase II trial, single arm,  open label | ORR 63% (PR 2/11, SD 4/11) |  |
| PRRT  177Lu-DOTATATE or  90Y-DOTATOC  Lapa et al. [19] |  | DTC: 8 (progressive RAI  refractory)  MTC: 4 | Assess tumor  heterogenicity in  predicting PFS  and OS | Phase II trial, single arm,  open label | Mean PFS 221 days  Mean OS 450 days |  |
| mTOR Inhibitors | | | | | | |
| Everolimus  Lim et al. [20] | mTOR | Thyroid cancer (all subtypes):  38 | Disease control rate  (PR + SD > 12 weeks) | Phase II, single arm, open label | PR: 5% (2/38, one PTC patient and  one FTC)  SD: 76% | Mucositis, anorexia, abnormal, liver enzymes, acneiform rash |
| Everolimus  Hanna et al. [21] |  | DTC: 33  MTC: 10  ATC: 7 | PFS | Phase II, single arm, open label | DTC: Median PFS 12.9 months,  PR 1/38  MTC: Median PFS 13.1 months,  PR 1/10  ATC: Median PFS 2.2 months, PR 1/7 |  |
| **Immunotherapy** | | | | | | |
| Pembrolizumab  Mehnert et al. [22] | PD-1 receptor | DTC: 22 (Refractory to  standard therapy, with PD-L1  expression) | ORR | Phase 1b, single arm,  open label  (KEYNOTE-28 Trial) | ORR: 9%  PR: 2/22 (9%)  SD: 13/22 (59%)  Median PFS (PR+SD): 7 months | Diarrhea, fatigue, pruritis, rash,  colitis (grade 3 in one patient) |
| **Combination Therapies Under Investigation** | | | | | | |
| Cabozantinib and  Atezolizumab  NCT03170960 | Cabozantinib (see above),  Atezolizumab: PD-L1 | Multiple tumors,  including DTC that is locally  advanced or metastatic | Dose escalation:  maximum tolerated  Dose  Expansion: ORR | Phase I/II,  dose escalation/expansion,  open label, recruiting | Estimated End Date 12/2021  (clinicaltrials.gov) | N/A |
| Cabozantinib, Nivolumab,  and Ipilimumab  NCT03914300 | Cabozantinib (see above),  Nivolumab: PD-1,  Ipilimumab: CTLA-4 | DTC (RAI refractory  progressive after one prior  VEGFR therapy) | ORR | Phase II, single group  assignment, open label,  recruiting | Estimated End Date 1/2021  (clinicaltrials.gov) | N/A |
| Cediranib Maleate with or  without Lenalidomide  NCT01208051 | Cediranib: VEGFR,  Lenalidomide: CRL4CRBN  E3 ubiquitin ligase | DTC (unresectable progressive  RAI refractory disease) | Phase I: Maximum  tolerated dose  Phase II: PFS | Phase I/II, parallel assignment,  randomized, open label, active,  not recruiting | Estimated End Date 2/2020  (clinicaltrials.gov) | N/A |
| Lenvatinib and Denosumab  NCT03732495 | Lenvatinib (see above),  Denosumab: RANKL | DTC (RAI resistant with  bone metastasis) | Skeletal-related  event-free (multiple) | Phase II, single group  assignment, open label,  recruiting | Estimated End Date 6/2022  (clinicaltrials.gov) | N/A |
| Lenvatinib and  Pembrolizumab  (PD-1 Inhibitor)  NCT02973997 | Lenvatinib (see above),  Pembrolizumab:  (see above) | DTC (progressive RAI  refractory disease) | ORR | Phase II, single group  assignment, open label,  active not recruiting | Estimated End Date 9/2022  (clinicaltrials.gov) | N/A |

R**eferences**

1. Al-Jundi M, Thakur S, Gubbi S, et al. 2020 Novel targeted therapies for metastatic thyroid cancer—a comprehensive review. Cancers (Basel). MDPI AG.
2. Cohen, E.E.; Rosen, L.S.; Vokes, E.E.; Kies, M.S.; Forastiere, A.A.; Worden, F.P.; Kane, M.A.; Sherman, E.; Kim, S.; Bycott, P. Axitinib is an active treatment for all histologic subtypes of advanced thyroid cancer: Results from a phase II study. J. Clin. Oncol. 2008, 26, 4708. [CrossRef] [PubMed]
3. Capdevila, J.; Trigo, J.M.; Aller, J.; Manzano, J.L.; Adrián, S.G.; Llopis, C.Z.; Reig, Ò.; Bohn, U.; y Cajal, T.R.; Duran-Poveda, M. Axitinib treatment in advanced RAI-resistant di_erentiated thyroid cancer (DTC) and refractory medullary thyroid cancer (MTC). Eur. J. Endocrinol. 2017, 177, 309–317. [CrossRef] [PubMed]
4. Lim, S.M.; Chung, W.Y.; Nam, K.-H.; Kang, S.-W.; Lim, J.Y.; Kim, H.-G.; Shin, S.H.; Sun, J.-M.; Kim, S.-G.; Kim, J.-H. An open label, multicenter, phase II study of dovitinib in advanced thyroid cancer. Eur. J. Cancer 2015, 51, 1588–1595. [CrossRef] [PubMed]
5. Schlumberger, M.; Tahara, M.; Wirth, L.J.; Robinson, B.; Brose, M.S.; Elisei, R.; Habra, M.A.; Newbold, K.; Shah, M.H.; Ho_, A.O. Lenvatinib versus placebo in radioiodine-refractory thyroid cancer. N. Engl. J. Med. 2015, 372, 621–630. [CrossRef]
6. Bible, K.C.; Suman, V.J.; Molina, J.R.; Smallridge, R.C.; Maples, W.J.; Menefee, M.E.; Rubin, J.; Sideras, K.; Morris III, J.C.; McIver, B. E_cacy of pazopanib in progressive, radioiodine-refractory, metastatic di_erentiated thyroid cancers: Results of a phase 2 consortium study. Lancet Oncol. 2010, 11, 962–972. [CrossRef]
7. Brose, M.S.; Nutting, C.M.; Jarzab, B.; Elisei, R.; Siena, S.; Bastholt, L.; De La Fouchardiere, C.; Pacini, F.; Paschke, R.; Shong, Y.K. Sorafenib in radioactive iodine-refractory, locally advanced or metastatic differentiated thyroid cancer: A randomised, double-blind, phase 3 trial. Lancet 2014, 384, 319–328. [CrossRef]
8. Capdevila, J.; Iglesias, L.; Halperin, I.; Segura, A.; Martínez-Trufero, J.; Vaz, M.Á.; Corral, J.; Obiols, G.; Grande, E.; Grau, J.J. Sorafenib in metastatic thyroid cancer. Endocr. Relat. Cancer 2012, 19, e209. [CrossRef]
9. Bikas, A.; Kundra, P.; Desale, S.; Mete, M.; O’Keefe, K.; Clark, B.G.;Wray, L.; Gandhi, R.; Barett, C.; Jelinek, J.S. Phase 2 clinical trial of sunitinib as adjunctive treatment in patients with advanced di_erentiated thyroid cancer. Eur. J. Endocrinol. 2016, 174, 373–380. [CrossRef] [PubMed]
10. Carr, L.L.; Manko_, D.A.; Goulart, B.H.; Eaton, K.D.; Capell, P.T.; Kell, E.M.; Bauman, J.E.; Martins, R.G. Phase II study of daily sunitinib in FDG-PET–positive, iodine-refractory di_erentiated thyroid cancer and metastatic medullary carcinoma of the thyroid with functional imaging correlation. Clin. Cancer Res. 2010, 16, 5260–5268. [CrossRef] [PubMed]
11. Ravaud, A.; de la Fouchardière, C.; Caron, P.; Doussau, A.; Do Cao, C.; Asselineau, J.; Rodien, P.; Pouessel, D.; Nicolli-Sire, P.; Klein, M. A multicenter phase II study of sunitinib in patients with locally advanced or metastatic di_erentiated, anaplastic or medullary thyroid carcinomas: Mature data from the THYSU study. Eur. J. Cancer 2017, 76, 110–117. [CrossRef] [PubMed]
12. Leboulleux, S.; Bastholt, L.; Krause, T.; de la Fouchardiere, C.; Tennvall, J.; Awada, A.; Gómez, J.M.; Bonichon, F.; Leenhardt, L.; Sou_et, C. Vandetanib in locally advanced or metastatic di_erentiated thyroid cancer: A randomised, double-blind, phase 2 trial. Lancet Oncol. 2012, 13, 897–905. [CrossRef]
13. Falchook, G.S.; Millward, M.; Hong, D.; Naing, A.; Piha-Paul, S.; Waguespack, S.G.; Cabanillas, M.E.; Sherman, S.I.; Ma, B.; Curtis, M. BRAF inhibitor dabrafenib in patients with metastatic BRAF-mutant thyroid cancer. Thyroid 2015, 25, 71–77. [CrossRef]
14. Brose, M.S.; Cabanillas, M.E.; Cohen, E.E.; Wirth, L.J.; Riehl, T.; Yue, H.; Sherman, S.I.; Sherman, E.J. Vemurafenib in patients with BRAFV600E-positive metastatic or unresectable papillary thyroid cancer refractory to radioactive iodine: A non-randomised, multicentre, open-label, phase 2 trial. Lancet Oncol. 2016, 17, 1272–1282. [CrossRef]
15. Ho, A.L.; Grewal, R.K.; Leboeuf, R.; Sherman, E.J.; Pfister, D.G.; Deandreis, D.; Pentlow, K.S.; Zanzonico, P.B.; Haque, S.; Gavane, S. Selumetinib-enhanced radioiodine uptake in advanced thyroid cancer. N. Engl. J. Med. 2013, 368, 623–632. [CrossRef]
16. Iravani, A.; Solomon, B.; Pattison, D.A.; Jackson, P.; Ravi Kumar, A.; Kong, G.; Hofman, M.S.; Akhurst, T.; Hicks, R.J. Mitogen-Activated Protein Kinase Pathway Inhibition for Redi_erentiation of Radioiodine Refractory Di_erentiated Thyroid Cancer: An Evolving Protocol. Thyroid 2019, 29, 1634–1645. [CrossRef]
17. Budiawan, H.; Salavati, A.; Kulkarni, H.R.; Baum, R.P. Peptide receptor radionuclide therapy of treatment-refractory metastatic thyroid cancer using (90)Yttrium and (177)Lutetium labeled somatostatin analogs: Toxicity, response and survival analysis. Am. J. Nucl. Med. Mol. Imaging 2013, 4, 39–52.
18. Versari, A.; Sollini, M.; Frasoldati, A.; Fraternali, A.; Filice, A.; Froio, A.; Asti, M.; Fioroni, F.; Cremonini, N.; Putzer, D.; et al. Di_erentiated thyroid cancer: A new perspective with radiolabeled somatostatin analogues for imaging and treatment of patients. Thyroid 2014, 24, 715–726. [CrossRef]
19. Lapa, C.; Werner, R.A.; Schmid, J.S.; Papp, L.; Zsótér, N.; Biko, J.; Reiners, C.; Herrmann, K.; Buck, A.K.; Bundschuh, R.A. Prognostic value of positron emission tomography-assessed tumor heterogeneity in patients with thyroid cancer undergoing treatment with radiopeptide therapy. Nucl. Med. Biol. 2015, 42, 349–354.[CrossRef]
20. Lim, S.; Chang, H.; Yoon, M.; Hong, Y.; Kim, H.; Chung, W.; Park, C.; Nam, K.; Kang, S.; Kim, M. A multicenter, phase II trial of everolimus in locally advanced or metastatic thyroid cancer of all histologic subtypes. Ann. Oncol. 2013, 24, 3089–3094. [CrossRef]
21. 102. Hanna,G.J.; Busaidy,N.L.;Chau,N.G.;Wirth,L.J.; Barletta, J.A.;Calles,A.;Haddad,R.I.;Kraft, S.;Cabanillas,M.E.; Rabinowits, G. Genomic correlates of response to everolimus in aggressive radioiodine-refractory thyroid cancer: A phase II study. Clin. Cancer Res. 2018, 24, 1546–1553. [CrossRef]
22. Mehnert, J.M.; Varga, A.; Brose,M.S.; Aggarwal, R.R.; Lin, C.-C.; Prawira, A.; De Braud, F.; Tamura, K.; Doi, T.; Piha-Paul, S.A. Safety and antitumor activity of the anti–PD-1 antibody pembrolizumab in patients with advanced, PD-L1–positive papillary or follicular thyroid cancer. BMC Cancer 2019, 19, 196. [CrossRef] [PubMed]
